# Skin conductance responses in Major Depressive Disorder (MDD) under mental arithmetic stress

**DOI:** 10.1371/journal.pone.0213140

**Published:** 2019-04-03

**Authors:** Ah Young Kim, Eun Hye Jang, Kwan Woo Choi, Hong Jin Jeon, Sangwon Byun, Joo Yong Sim, Jae Hun Choi, Han Young Yu

**Affiliations:** 1 Bio-Medical IT Convergence Research Department, Electronics and Telecommunications Research Institute(ETRI), Gajeong-Ro, Yoseong-Gu, Daejeon, Rep. of Korea; 2 Department of Psychiatry, Depression Center, Samsung Medical Center, Sungkyunkwan University School of Medicine, Irwon-ro, Gangnam-gu, Seoul, Rep. of Korea; 3 Department of Electronics Engineering, Incheon National University, Incheon, Korea; Universita degli Studi di Pisa, ITALY

## Abstract

Depressive symptoms are related to abnormalities in the autonomic nervous system (ANS), and physiological signals that can be used to measure and evaluate such abnormalities have previously been used as indicators for diagnosing mental disorder, such as major depressive disorder (MDD). In this study, we investigate the feasibility of developing an objective measure of depressive symptoms that is based on examining physiological abnormalities in individuals when they are experiencing mental stress. To perform this, we recruited 30 patients with MDD and 31 healthy controls. Then, skin conductance (SC) was measured during five 5-min experimental phases, comprising baseline, mental stress, recovery from the stress, relaxation, and recovery from the relaxation, respectively. For each phase, the mean amplitude of the skin conductance level (MSCL), standard deviations of the SCL (SDSCL), slope of the SCL (SSCL), mean amplitude of the non-specific skin conductance responses (MSCR), number of non-specific skin conductance responses (NSCR), and power spectral density (PSD) were evaluated from the SC signals, producing 30 parameters overall (six features for each phase). These features were used as input data for a support vector machine (SVM) algorithm designed to distinguish MDD patients from healthy controls based on their physiological responses. Statistical tests showed that the main effect of task was significant in all SC features, and the main effect of group was significant in MSCL, SDSCL, SSCL, and PSD. In addition, the proposed algorithm achieved 70% accuracy, 70% sensitivity, 71% specificity, 70% positive predictive value, 71% negative predictive value in classifying MDD patients and healthy controls. These results demonstrated that it is possible to extract meaningful features that reflect changes in ANS responses to various stimuli. Using these features, detection of MDD was feasible, suggesting that SC analysis has great potential for future diagnostics and prediction of depression based on objective interpretation of depressive states.

## Introduction

Major depressive disorder (MDD) is a disabling illness associated with feelings of depression, hopelessness, pessimism, low self-esteem, and despair. It is an extremely serious condition, with ~16% of cases having a lifetime prevalence and ~60% being of clinical severity [1–3]. The characteristics of the disorder mean it can cause significant problems in regard to work performance and can also increase the economic burden of society [4,5]. Currently, diagnosis of depression relies primarily on clinicians’ rating scales and specialized questionnaires, such as the Diagnostic and Statistical Manual of Mental Disorders (DSM) [6]. However, the accuracy of this approach is influenced by clinicians’ subjective evaluations and interpretations of patient interviews. Furthermore, diagnosis based on the DSM categorizes mental illness as a state in which the boundaries that distinguish moods are ambiguous and overlapping [7,8]. Consequently, there is a need for reliable diagnostic tools that can be used to assess and predict depressive symptoms easily and in an objective manner, taking psychophysiology into account.

Physiological signals are potential candidates for objective measures of MDD diagnosis, as shown by previous studies that have evaluated autonomic nervous system (ANS) dysfunction in MDD patients by analyzing their physiological signals [9–11]. In fact, some physiological signals have been tested as clinical evaluation measures, but none have yet been used for clinical purposes. Nonetheless, recent studies have demonstrated the possibility that physiological signals can be used as biomarkers for depressive symptoms [12,13]. For example, studies have shown that changes in the clinical status of MDD patients can be detected by monitoring electrocardiograms, respiration [14], pupillary dynamics [15], and electroencephalograms [16]. In addition, skin conductance (SC) is also a compelling candidate as an objective measure of MDD, as it is a peripheral indicator of sympathetic arousal in response to changes in emotional state [17]. Generally, MDD patients show lower levels of SC than do healthy controls. For example, Lacono et al. reported that patients with unipolar affective disorder showed decreased phasic and tonic responses regarding SC than do controls [18]. Moreover, Myslobodsky et al. [19] examined bilateral SC in depressive patients during visual and verbal tasks and a tone habituation sequence, finding that, in patients with endogenous depression, SC was higher in the left hand than in the right hand, regardless of the given conditions. Additionally, patients with reactive depression showed higher SC in their left hand during the verbal task and tone habituation sequence, but the opposite result was observed during the visual task. Similarly, Williams et al. [20] observed differences in SC between individuals with affective disorder and normal controls when the results for a verbal task were compared, but no differences were observed for the results of a visual task or between individuals with unipolar and bipolar affective disorders, except in regard to psychomotor status (retarded/non-retarded distinction). Finally, Greco et al. suggested that phasic SC during emotional stimulation can be a suitable indicator of mood status in patients with bipolar disorder [21].

Despite the above findings and several years of research, it remains difficult to find reliable and consistent results from previous studies of depressive disorder, largely because MDD presents heterogeneous profiles with respect to clinical symptoms, clinical severity, age of onset, duration of episodes, clinical progress, and possession of other disorders (comorbidity) [22].

The effect of stress on depression is an emerging topic in psychiatric and psychological research, and many clinical studies have shown that depression and stress are related [23–26]. For example, one study showed that, in patients with MDD, exposure to psychological stress reduced sensitivity to the anti-inflammatory properties of glucocorticoids but, in healthy controls, such exposure increased this sensitivity [27]. Furthermore, for individuals experiencing stress, depression has been shown to induce changes in cognition such that the stress-causing situation is perceived as severe and unsolvable [28]; to delay recovery from stressors as a result of the negative cognitive style fostered; and to diminish heart rate recovery from laboratory stress [29]. For this reason, in this study, we hypothesized that there is a difference between MDD patients and healthy controls regarding the level of perceived arousal before, during, and after an episode of mental stress that is then followed by a relaxation task. A perception-based task can be beneficial for negotiating the differences in heterogeneous reactivity reported across many previous depression studies. Therefore, in this study, we are proposing a method of using SC to measure the physiological manifestations of psychological processes induced by stress and relaxation tasks. This would allow individuals with MDD to be distinguished from healthy individuals. To verify this, we use a support vector machine (SVM) to evaluate the feasibility of SC features in terms of detecting MDD.

## Method

### Subjects

Thirty patients with MDD and 31 healthy controls who had no history of psychiatric disorder participated in the current study. Patients were diagnosed by a senior psychiatrist, and those who scored ≥ 16 on the Hamilton Depression Rating Scale (HAM-D; comprising 21-items) were allocated to the MDD group. We also used the stress response inventory (SRI) and perceived stress scale (PSS) to evaluate depressive symptoms in the participants. All subjects were informed of the purpose of the experiment and the methods involved, and they then provided written informed consent. All participants were paid approximately $50 in return for their participation. This study was approved by the Institutional Review Board of Samsung Medical Center of Seoul, Korea (No. 2015-07-151) and performed in accordance with the relevant guidelines.

### Experimental paradigm

**Fig 1:**
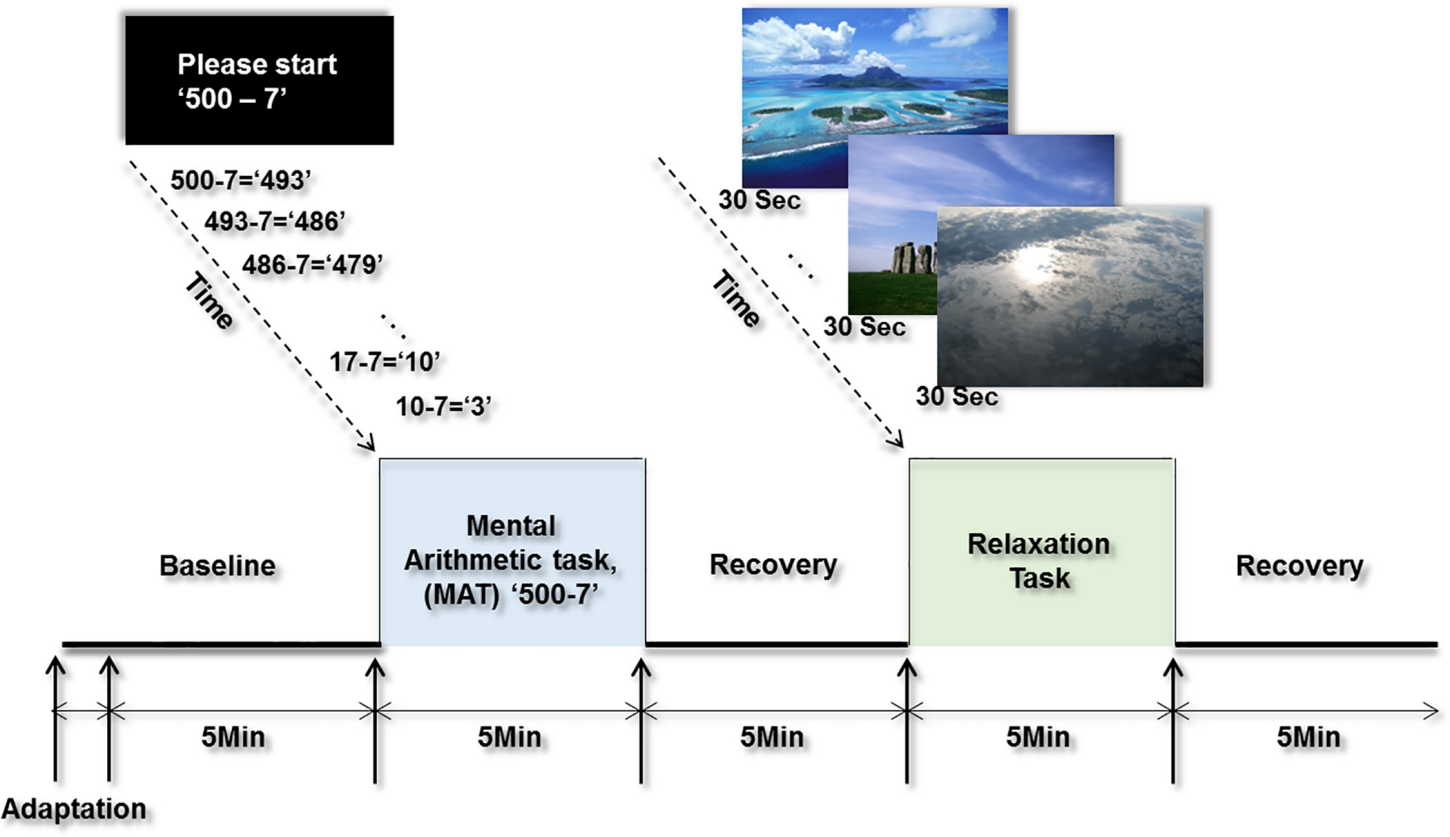
Experimental protocol. SC signals were obtained across five phases. Each phase had a duration of 5-min.

The study protocol comprised five phases, and each phase persisted for 5 min: the baseline, a mental stress task, recovery from the mental stress task, a relaxation task, and recovery from the relaxation task; the experimental paradigm is shown in Fig 1. Both the mental arithmetic task (MAT) [30] and relaxation task [31,32] were designed to evaluate the differences between the MDD patients and the healthy controls regarding the reactivity of their ANSs. This approach accords with previous studies that have utilized MATs as standard stressors for detecting changes in participants’ ANS [33–35]. The MAT task for this study gradually increased the subjects’ mental load by asking them to begin at the number 500 and to perform continuous subtraction in units of seven. Then, during the relaxation task, 10 consecutive pictures of natural landscapes were shown to the subjects, which allowed us to investigate differences in ANS responses during recovery from the stressor [31].

### Physiological signal measurements

Before beginning the measurement, subjects were asked to sit in an armchair, after which a clinical assistant provided them with a detailed explanation of the experimental procedures. They were also given an adaptation period prior to the start of the experiment. Then, SC signals were measured and recorded throughout the experiment, assessing different patterns of responses experienced by subjects during the five phases.

The physiological signals were recorded using ProComp Infiniti (SA7500, Computerized Biofeedback System, Thought Technology, Ltd., Canada). For each subject, SC sensors were attached to the distal phalanges of the index and ring fingers of the left hand to measure the skin’s sweat secretion responses, which was conducted at a fixed sampling frequency of 256 Hz.

### Signal pre-processing and feature extraction

**Fig 2:**
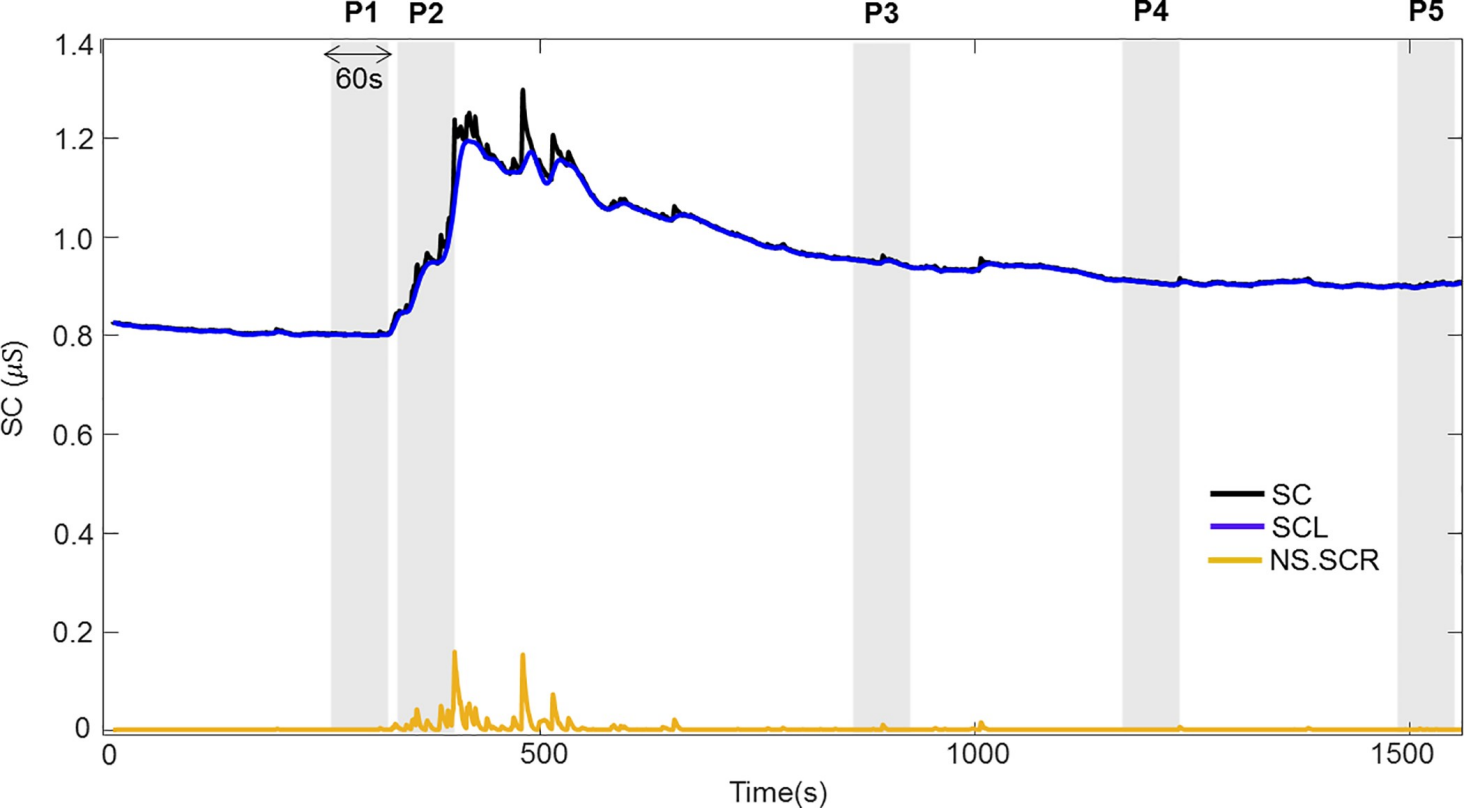
Decomposition of the SC signal. The SC signal (top black line) was decomposed into SCL (tonic component, blue) and SCR (phasic component, yellow) using the cvxEDA model. SC features were calculated from the final 60-s period of the baseline phase (P1), the first 60-s period of the MAT task (P2), and the final 60-s periods of the first recovery (P3), relaxation task (P4), and the second recovery (P5) phases, respectively.

SC was analyzed using MATLAB R2017b (MathWorks, Inc., MA, USA). Artifacts, such as gestures and body movements, which could distort the data, were removed before analysis of the SC physiological responses. Then, the SC signals were decomposed using a convex optimization model (cvxEDA [36]). The cvxEDA model was adapted to each time series after Z-score normalization. Specifically, the cvxEDA model describes SC as the sum of three components: tonic component, phasic component, and additive white Gaussian noise. The tonic component (skin conductance level; SCL) represents the base level of the signal, whereas the phasic component (skin conductance responses; SCR) reflects a direct response to an external stimulus (1–5 sec after stimulus onset). The non-specific SCR (NS.SCR) that appears post-stimuli represents the number of SCRs within a period of time. In the present study, features extracted from SCL and NS.SCRs were calculated based on 60-sec non-overlapping time windows for P1–P5, respectively (Fig 2). The features for P1 were calculated based on the last 60-sec period; for P2 (the MAT task), the first 60-sec period was selected, which reflected responses to the stimulus; and for P3, P4, and P5, the final 60-sec periods of each phase were used, which allowed the participants sufficient time to recover. Fig 2 shows the overall SC signal (black line) decomposed into SCL (blue line) and NS.SCR (yellow line). The SCL presents as a slowly-varying low-frequency signal, whereas the NS.SCR is depicted as a rapidly varying high-frequency signal. Three SCL features were obtained in time-domain: the mean amplitude of the SCL (MSCL), the standard deviations of the SCL (SDSCL), and the slope of the SCL (SSCL). The features of the NS.SCRs were the mean amplitude of the NS.SCRs (MSCR), and the number of NS.SCRs (NSCR). The same 60-sec segments selected to compute time-domain features were used for time-invariant spectral analysis. Here, SC signals were down-sampled to 2 Hz prior to spectral analysis. For time-invariant spectral analysis, power spectral density (PSD) analysis of the SC signals was also performed, using Welch’s periodogram methods with 50% data overlap (for a detailed description, see [37]). Thus, a total of 30 parameters (six features from each of the five periods) were calculated.

### Statistical analyses

Statistical analyses were performed using MATLAB R2017b and R software 3.5.1 (The R Foundation for Statistical Computing, Vienna, Austria). The Shapiro-Wilk Test was performed to test the normality assumption. Since age, SRI, PSS, and HAM-D scores were found not to be normally distributed, we used a non-parametric Mann-Whitney U test to compare MDD patients and healthy controls. A chi-square test was performed to compare gender. MSCL, SDSCL, SSCL, MSCR, NSCR, and PSD did not meet the normality assumption, and, therefore, to test the effects of group and task, we conducted non-parametric analysis of longitudinal data in factorial designs using the R-software “nparLD” package [38]. The Bonferroni method was used to correct *P* values for multiple comparisons.

### Classification

All extracted features were used to classify differences between the MDD patients and the healthy controls in terms of their physiological characteristics. To evaluate the performance of the classifier, we applied the leave-one-out (LOO) procedure using an SVM-based classifier. The SVM is a supervised machine learning method and is used to find an optimal separating hyperplane for discrimination. The kernel used in this study was the polynomial model, which allowed learning of non-linear models, as shown in Eq (1) [39]. The polynomial kernel represents the similarity of training data in a feature space over polynomials of the original data. The SVM classifier performance is determined by the parameters *γ*, *r*, and *d*. We selected the best combination, *γ* = 1, *r* = 0.2, and *d* = 3, using a grid search.

$$K\;(x_{i},x_{j})={\left(\gamma x_{i},x_{j}+r\right)}^{d}$$ (1)

Within the LOO procedure, the features were normalized by subtracting the median value and dividing by the median absolute deviation. The LOO was repeated N = 61 times, and the performances were averaged. Classification results were represented using accuracy, sensitivity, specificity, positive predictive value (PPV), and negative predictive value (NPV). The PPV was defined as the proportion of correctly classified MDD patients, and NPV was defined as the proportion of correctly classified healthy controls. All analyses were performed using MATLAB R2017b with the additional toolbox “LIMSVM” [40].

## Results

### Demographic and clinical characteristics of the MDD patients and healthy controls

Table 1 shows the statistical characteristics of gender, age, and psychological parameters such as SRI, PSS, and HAM-D scores, of the MDD patients and healthy controls. The MDD group included 12 males and 18 females, with an average age of 42.5 years. Meanwhile, the control group included 13 males and 18 females, with an average age of 43.7 years. There were no significant differences between the two groups regarding gender (p = 1.00) or age (p = 0.79). However, we observed significant differences between the groups regarding SRI (p < 0.001), PSS (p < 0.05), and HAM-D (p < 0.001) scores.

**Table 1:** Demographic and clinical characteristics of subjects.

Characteristics	MDD (N = 30)	Control (N = 31)	*P*
Gender, male (%) ^¶^	12 (40%)	13(42%)	1.00
Age, years^ª^	42.5 (19,70)	43.7 (20,77)	0.79
SRI^ª^	69.8 (15,133)	12 (0,41)	< 0.001***
PSS^ª^	17.8 (0.34)	15.4 (0, 22)	0.049*
HAM-D^ª^	19.48 (11,31)	1.6 (0, 6)	< 0.001***

Abbreviations: SRI, Stress Response Inventory; PSS, Perceived Stress Scale; HAM-D, Hamilton Depression Rating Scale.

^¶^ Chi-square test was performed.

^ª^ The factors were compared using Mann-Whitney U test (*p < 0.05, ***p < 0.001).

### Comparison of the features of the MDD patients and healthy controls

The mean values of MSCL, SDSCL, SSCL, MSCR, NSCR, and PSD for each respective phase in the MDD and control groups are shown in Table 2. Comparing the SC features of the two groups, all six features were lower in the MDD patients than in the healthy controls for all phases.

**Table 2:** Mean (SD) values of the SC features of the MDD patients and the healthy controls during P1-P5.

MSCL (μS)					
	P1	P2	P3	P4	P5
	Mean (SD)	Mean (SD)	Mean (SD)	Mean (SD)	Mean (SD)
MDD	0.58 (0.46)	0.79 (0.69)	1.01 (0.96)	0.88 (0.78)	0.86 (0.82)
Control	1.14 (1.64)	1.55 (2.01)	1.58 (2.01)	1.48 (1.95)	1.58 (1.95)
SDSCL (μS)					
	P1	P2	P3	P4	P5
	Mean (SD)	Mean (SD)	Mean (SD)	Mean (SD)	Mean (SD)
MDD	0.00 (0.00)	0.06 (0.14)	0.00 (0.01)	0.00 (0.01)	0.00 (0.01)
Control	0.01 (0.03)	0.19 (0.33)	0.01 (0.01)	0.01 (0.02)	0.03 (0.09)
SSCL (μS)					
	P1	P2	P3	P4	P5
	Mean (SD)	Mean (SD)	Mean (SD)	Mean (SD)	Mean (SD)
MDD	0.00 (0.00)	0.01 (0.02)	0.00 (0.01)	0.00 (0.01)	0.00 (0.00)
Control	0.00 (0.01)	0.03 (0.05)	0.01 (0.01)	0.01 (0.01)	0.01 (0.01)
NSCR					
	P1	P2	P3	P4	P5
	Mean (SD)	Mean (SD)	Mean (SD)	Mean (SD)	Mean (SD)
MDD	0.87 (1.50)	4.47 (4.37)	1.83 (2.21)	1.37 (1.85)	1.57 (2.40)
Control	1.58 (2.88)	6.65 (5.8)	2.26 (2.90)	1.94 (2.05)	3.03 (3.16)
MSCR (μS)					
	P1	P2	P3	P4	P5
	Mean (SD)	Mean (SD)	Mean (SD)	Mean (SD)	Mean (SD)
MDD	0.02 (0.03)	0.09 (0.14)	0.05 (0.09)	0.03 (0.06)	0.04 (0.06)
Control	0.08 (0.22)	0.27 (0.45)	0.11 (0.23)	0.09 (0.14)	0.17 (0.31)
PSD					
	P1	P2	P3	P4	P5
	Mean (SD)	Mean (SD)	Mean (SD)	Mean (SD)	Mean (SD)
MDD	2.50 (4.18)	6.98 (11.47)	9.04 (14.98)	6.31 (10.62)	6.68 (13.31)
Control	17.74 (51.32)	51.07 (128.02)	32.42 (88.65)	28.2 (74.37)	32.18 (81.53)

Abbreviations: MSCL, mean amplitude of the SCL; SDSCL, standard deviations of the SCL; SSCL, slop of the SCL; NSCR, the number of NS.SCRs; MSCR, mean amplitude of the NS.SCRs; PSD, power spectral density of SC

**Fig 3:**
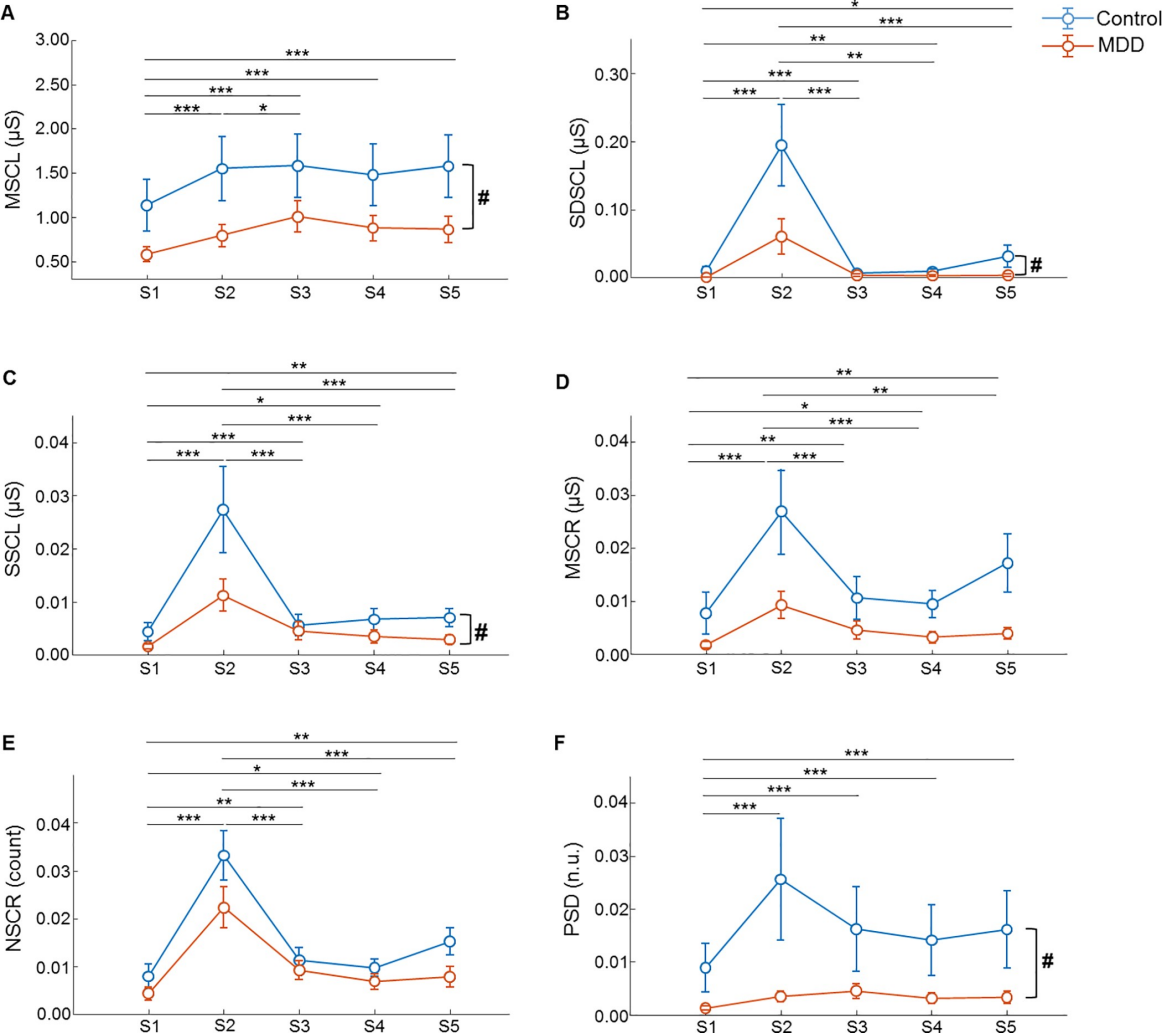
**Mean ± SE of (A) MSCL, (B) SDSCL, (C) SSCL, (D) MSCR, (E) NSCR, and (F) PSD.** Effects of group and task on the (A)-(F) features of the MDD patients (N = 30) and the healthy controls (N = 31) were analyzed using the non-parametric equivalent of a repeated-measures ANOVA (#p < 0.05). Post-hoc comparisons of tasks were corrected using the Bonferroni method (*p < 0.05, **p < 0.01, ***p < 0.001).

We tested the effects of group and task using the non-parametric equivalent of a repeated-measures ANOVA. Consequently, we determined that MSCL (F = 5.84, p < 0.05), SDSCL (F = 4.70, p < 0.05), SSCL (F = 4.29, p < 0.05), and PSD (F = 5.93, p < 0.05) were significantly affected by group, but that MSCR (F = 3.79, p = 0.052) and NSCR (F = 2.78, p = 0.095) were not (Table 3 and Fig 3). All features were significantly affected by the main effect of task (p < 0.001; Table 3). However, there was no significant interaction between group and task (Table 3). The results of post-hoc comparisons between tasks are shown in Table 4 and Fig 3. The MSCL from P1 was significantly lower than those from P2-P5 (p < 0.001). The MSCL was significantly lower in P2 than P3 (p < 0.05). The SDSCL from P1 was significantly lower than those from the other phases (P1 and P2: p < 0.001; P1 and P3: p < 0.001; P1 and P4: p < 0.01; P1 and P5: p < 0.05). Meanwhile, the SDSCL from P2 was significantly higher than those from P3, P4, and P5 (p < 0.001). The SSCL from P1 was significantly lower than those from the other phases (P1 and P2: p < 0.001; P1 and P3: p < 0.001; P1 and P4: p < 0.05; P1 and P5: p < 0.01), while the SSCL from P2 was significantly higher than those from P3, P4, and P5 (p < 0.001). The MSCR from P1 was significantly lower than those from the other phases (P1 and P2: p < 0.001; P1 and P3: p < 0.01; P1 and P4: p < 0.05; P1 and P5: p < 0.01), while the MSCR from P2 was significantly higher than those from P3 (p < 0.001), P4 (p < 0.001), and P5 (p < 0.01). The NSCR from P1 was significantly lower than those from the other phases (P1 and P2: p < 0.001; P1 and P3: p < 0.01; P1 and P4: p < 0.05; P1 and P5: p < 0.01), while the NSCR from P2 was significantly higher than those from P3, P4, and P5 (p < 0.001). The PSD from P1 was significantly lower than those from the other phases (p < 0.001).

**Table 3:** Effects of group and task on SC features. Statistical analyses were performed using the non-parametric equivalent of a repeated-measures ANOVA through the R statistics package “nparLD.”. Group was used as the between-subjects factor and task as the within-subject factor (*p < 0.05, ***p < 0.001).

Feature	Group effect (MDD vs. control)		Task effect (5 phases)		Interaction (group × task)	
	F	p	F	p	F	P
MSCL	5.87	0.015*	28.81	< 0.001***	0.90	0.436
SDSCL	4.70	0.030*	38.55	< 0.001***	1.03	0.383
SSCL	4.29	0.038*	28.10	< 0.001***	1.09	0.358
MSCR	3.79	0.052	16.53	< 0.001***	0.80	0.519
NSCR	2.78	0.095	28.63	< 0.001***	1.67	0.158
PSD	5.93	0.015*	25.47	< 0.001***	0.75	0.527

**Table 4:** Post-hoc pairwise comparisons. Post-hoc pairwise comparisons between tasks were corrected using the Bonferroni method (*p < 0.05, **p < 0.01, ***p < 0.001).

Pairwise comparison	Bonferroni adjusted *P*					
	MSCL	SDSCL	SSCL	MSCR	NSCR	PSD
P1 vs. P2	< 0.001***	< 0.001***	< 0.001***	< 0.001***	< 0.001***	< 0.001***
P1 vs. P3	< 0.001***	< 0.001***	< 0.001***	0.009**	0.005**	< 0.001***
P1 vs. P4	< 0.001***	0.005**	0.034*	0.043*	0.072*	< 0.001***
P1 vs. P5	< 0.001***	0.024*	0.008**	0.002**	0.002**	< 0.001***
P2 vs. P3	0.025*	< 0.001***	< 0.001***	< 0.001***	< 0.001***	1.000
P2 vs. P4	1.000	< 0.001***	< 0.001***	< 0.001***	< 0.001***	1.000
P2 vs. P5	1.000	< 0.001***	< 0.001***	0.005**	< 0.001***	1.000
P3 vs. P4	0.682	1.000	1.000	1.000	1.000	1.000
P3 vs. P5	1.000	1.000	1.000	1.000	1.000	1.000
P4 vs. P5	1.000	1.000	1.000	1.000	1.000	1.000

We evaluated whether the features extracted from the SC signals were capable of distinguishing the MDD and control groups using an SVM (for further details, see the Methods section). Table 5 shows the performance results of the SVM classifier with selected features. The best performance, with 70.49% accuracy, 70.00% sensitivity, 70.97% specificity, 70.00% PPV, and 70.97% NPV, was achieved using the MSCL, SDSCL, SSCL, and NSCR features, demonstrating that MDD patients can be distinguished from healthy controls using features from SC signals.

**Table 5:** Performance measures of the SVM classifier based on input features.

Input features	Number	ACC (%)	SE (%)	SP (%)	PPV (%)	NPV (%)
MSCL, SDSCL	10	54.10	52.63	56.52	66.67	41.94
MSCL, SDSCL, SSCL	15	63.93	62.50	65.52	66.67	61.29
MSCL, SDSCL, NSCR	15	67.21	63.89	72.00	76.67	58.06
**MSCL, SDSCL, SSCL, NSCR**	**20**	**70.49**	**70.00**	**70.97**	**70.00**	**70.97**
MSCL, SDSCL, SSCL, MSCR, NSCR	25	68.85	67.74	70.00	70.00	67.74
MSCL, SDSCL, SSCL, NSCR, PSD	25	67.21	66.67	67.74	66.67	67.74
MSCL, SDSCL, SSCL, MSCR, NSCR, PSD	30	67.21	67.86	66.67	63.33	70.97

Abbreviations: ACC, accuracy; SE, sensitivity; SP, specificity.

## Discussion

We have explored the feasibility of distinguishing between MDD patients and healthy controls based on patterns in ANS dynamics produced in response to stimulation. To examine this, we used an experimental protocol that included baseline, a mental stress task, recovery from the mental stress task, a relaxation task, and recovery from the relaxation task. The main finding is that SC features measured during arousal and recovery can distinguish MDD patients from healthy controls, suggesting that SC features may represent biomarkers for MDD.

First, we tested whether our experimental protocol for SC features could reflect changes in ANS activity. To perform this, participants’ SC signals were decomposed into tonic and phasic components using through the cvxEDA model, and then six SC features were extracted using time-frequency analyses. The responses of all SC features were lower in the MDD patients than in the healthy controls (Table 2 and Fig 3). Also, the change in the SC response between the protocol phases was less in the MDD patients than in the healthy controls. These results are consistent with previous studies, which found that SC arousal levels were typically lower in subjects with depression [41–46], suggesting that the reactivity of SC features during arousal and recovery phases could be used to distinguish depressed and non-depressed subjects.

The effects of group and task on the SC features were statistically tested (Tables 3 and 4 and Fig 3). Notably, the significant increases observed in all six features during the MAT task indicated that the stress task had successfully induced changes in sympathetic activity [34]. In addition, the SC features for the recovery from stress phase (P3) were significantly lower than those for the stress task phase (P2), with the exception of the MSCL and PSD. However, the SC features for the relaxation task (P4) and the recovery from the relaxation (P5) phases were not significantly different from those of P3, suggesting that the relaxation task did not induce further sympathetic relaxation. These results were not consistent with previous studies, in which relaxation tasks were determined to facilitate recovery from stress [32]. Considering this, it is likely that the recovery from the stress phase (P3) was not sufficiently long for subjects to completely recover from the mental stress, which makes comparisons to subsequent P4 and P5 phases problematic. Interestingly, in the healthy controls, all features were higher in the recovery from the relaxation phase (P5) than in the relaxation phase (P4), which is consistent with previous findings that natural scenery increased ANS activity and improved mood and self-esteem [47]. Our results also demonstrated that the relaxation task did not increase ANS activity in the MDD patients, suggesting that this task may help to distinguish responses in ANS activity between MDD and healthy subjects.

Finally, we applied an SVM algorithm to detect MDD patients. In previous studies, several well–known machine learning algorithms have been used in attempts to determine an optimal method of identifying the ANS patterns of MDD patients and controls. For example, Sun et al. [48] applied logistic regression analysis to HRV features to differentiate 44 MDD and 47 healthy control subjects and achieved a sensitivity and specificity of 80% and 79%, respectively. Liao et al. [49] distinguished 20 normal and 20 depressed subjects with 81% accuracy using EEG signals and an SVM classifier. The performance in the current study (70% accuracy, 70% sensitivity, and 71% specificity) is relatively low compared to these previous studies. However, some of these studies lacked descriptions of their validation methods. Also, classification of MDD using SC signals is rarely studied, which makes direct comparisons difficult. In a future study, we will attempt to improve the model performance by including various SC features.

The limitation of this study is its small sample size. The sample size used in this study may not be optimal for reducing variances in accuracy, sensitivity, and specificity of classification. We are currently recruiting more subjects to expand our findings and believe that these efforts can help us to develop a tool for objectively diagnosing depression.

## Conclusion

We demonstrated that SC features measured in various states of ANS activity were highly relevant to depressive symptoms, suggesting that these physiological features can be used as suitable bio-markers for discriminating MDD. These results can contribute to the development of a new technique for diagnosing and predicting depression, such as through the use of a wearable system that monitors SC signals during various arousal and recovery states in naturalistic environments.

## Supporting information

S1 File Dataset of skin conductance (SC) features in major depressive disorder patients and healthy controls. (CSV)
